# Clinical outcomes of T4a papillary thyroid cancer with recurrent laryngeal nerve involvement: a retrospective analysis

**DOI:** 10.1038/s41598-021-86226-x

**Published:** 2021-03-23

**Authors:** Han-Seul Na, Hyun-Keun Kwon, Sung-Chan Shin, Yong-Il Cheon, Myeonggu Seo, Jin-Choon Lee, Eui-Suk Sung, Minhyung Lee, In-Joo Kim, Bo Hyun Kim, Byung-Joo Lee

**Affiliations:** 1 Department of Otolaryngology-Head and Neck Surgery, College of Medicine, Pusan National University and Medical Research Institute, Pusan National University Hospital, 179 Gudeok-ro, Seo-gu, Busan, 49241 South Korea; 2 Department of Otorhinolaryngology-Head and Neck Surgery, College of Research Institute for Convergence of Biomedical Science and Technology, Pusan National University Yangsan Hospital, Yangsan, South Korea; 3 Division of Endocrinology and Metabolism, Department of Internal Medicine, Biomedical Research Institute, Pusan National University Hospital, Busan, South Korea

**Keywords:** Endocrinology, Oncology, Risk factors

## Abstract

Preoperative vocal cord palsy (VCP) may indicate locally invasive papillary thyroid cancer (PTC); using this relationship, we evaluated the clinical outcomes and risk factors for recurrence in post-thyroidectomy T4a PTC patients with recurrent laryngeal nerve (RLN) involvement. We retrospectively investigated thyroidectomy patients, recorded their clinical factors, recurrence rate, and pathological findings, and analysed the relationship between recurrence rate and clinical factors. Of 72 patients, 37 (51%) had preoperative VCP and 35 (49%) had normal preoperative vocal cord movement with confirmed intraoperative RLN invasion. Tracheal and esophageal invasion was observed in 13 (18%) and 15 (21%) patients, respectively. Thyroid cancer recurred in 18 (25%) patients over 58 months, resulting in 2 (3%) deaths. Recurrence was not associated with surgical extent, organ invasion, enlarged tumour size, or lymph node infiltration (*p* > 0.05). The recurrence rate was significantly higher in patients with positive resection margins (*p* < 0.05). T4a PTC patients with RLN involvement showed a poor prognosis. The recurrence rate was not affected by preoperative VCP, intraoperative detection of RLN invasion, nerve resection, nerve preservation by shaving, lymph node metastasis, or tracheal or esophageal invasion. The most important prognostic factor for recurrence was a positive resection margin.

## Introduction

Papillary thyroid carcinoma (PTC) is the most common type of thyroid cancer. Differentiated thyroid cancer, especially papillary microcarcinoma, has an excellent prognosis with very low mortality^1^. However, according to the American Thyroid Association guidelines, PTC patients with macroscopic tumour invasion, incomplete resection, distant metastasis, and thyroglobulinemia are classified as a high-risk group for poor prognoses^2^.

The invasion of adjacent structures by PTC, with extrathyroidal extension, is termed locally invasive thyroid cancer, and it presents differently depending on the anatomical structures it invades, which influences the clinical presentation and therapeutic consequences^3,4^. In this regard, invasion of the recurrent laryngeal nerve (RLN) is one of the main predictors of poor prognosis in PTC patients; in a previous study, tumour recurrence occurred 3 times higher in RLN-involved patients than control-group^5^. RLN invasion can affect the surrounding vital organs, such as, trachea, and/or esophagus, which increases the range of surgery and worsens the prognosis^3^. For this reason, RLN invasion corresponds to a grade of T4a as per the American Joint Committee on Cancer classification.

It is possible to predict RLN invasion in PTC patients by checking for vocal cord palsy (VCP) before surgery. Previous studies reported that only 1–3% of patients with a thyroid tumour exhibited preoperative VCP^6,7^. However, even if vocal cord movements are normal before surgery, RLN invasion may be observed during the operation. There are controversies regarding whether the RLN should be resected when its involvement is suspected preoperatively or confirmed intraoperatively. When VCP is observed before surgery, the RLN tends to be sacrificed in the majority of cases. In contrast, when VCP is not observed preoperatively, there is a tendency to attempt to preserve the nerve as much as possible; however, the tumour may not be completely removed with this approach, resulting in a higher risk of recurrence.

The thyroid cancer recurrence rate has been reported to be 31.4% in cases with RLN involvement^5^. Nevertheless, there is a lack of studies wherein potential predictors of PTC recurrence after surgery in patients with RLN involvement are evaluated. Therefore, our study aimed to evaluate the clinical outcomes and risk factors associated with thyroid cancer recurrence after thyroidectomy in T4a PTC patients with RLN involvement.

## Results

Table 1 describes the characteristics of all our patients. Eighteen patients (25.0%) exhibited confirmed recurrence during the follow-up period. The sites of recurrence included: 6 (33.3%) patients on the central neck, 10 (55.6%) on the lateral neck, 7 (38.9%) with distant recurrence, and 5 (27.8%) with recurrence at multiple sites.

**Table 1:** Clinical characteristics of 72 patients with RLN invasion.

Characteristics		Overall	Recurrence	No recurrence	*p* value	* * p* < 0.05
n		72	18	54		
f/u time month (median [IQR])		58.00 [30.25 ~ 83.00]	38.50 [31.00, 59.00]	64.50 [28.75, 93.00]	0.091	
Sex (%)	F	56 (77.8)	11 (19.64)	45 (80.36)	0.098	
	M	16 (22.2)	7 (43.75)	9 (56.25)		
Age (mean (SD))		57.59 (14.17)	57.52 (14.47)	57.61 (14.20)	0.982	
Height (mean (SD))		159.48 (7.86)	161.75 (8.16)	158.71 (7.68)	0.176	
Body weight (mean (SD))		61.63 (11.89)	65.43 (15.82)	60.34 (10.08)	0.213	
BMI (mean (SD))		24.12 (3.38)	24.81 (4.07)	23.89 (3.12)	0.390	
Operative method (%)	HT	1 (1.4)	0 (0.0)	1 (100)	0.836	
	TT /c CND	43 (59.7)	10 (23.26)	33 (76.74)		
	TT /c CND /c LND	28 (38.9)	8 (28.57)	20 (71.43)		
RAI dose (%)	< 150	17 (26.2)	4 (23.53)	13 (76.47)	1.000	
	≥ 150	48 (73.8)	13 (27.08)	35 (72.92)		
Tg level post ablation (%)	≤ 1	22 (34.4)	3 (13.64)	19 (86.36)	0.137	
	> 1	42 (65.6)	14 (33.33)	28 (66.67)		
Pre op. VCP (%)	yes	37 (51.4)	10 (27.03)	27 (72.97)	0.788	
	no	35 (48.6)	8 (22.86)	27 (77.14)		
RLN direction (%)	Rt	32 (44.4)	8 (25.00)	24 (75.00)	1.000	
	Lt	39 (54.1)	10 (25.64)	29 (74.36)		
Tumor size (%)	≤ 20 mm	39 (54.2)	7 (17.95)	32 (82.05)	0.175	
	> 20 mm	33 (45.8)	11 (33.33)	22 (66.67)		
Resection margin (%)	clear	39 (54.2)	5 (12.82)	34 (87.18)	0.014	*
	involved	33 (45.8)	13 (39.39)	20 (60.61)		
Intra op. RLN resection (%)	yes	47 (65.3)	15 (31.91)	32 (68.09)	0.088	
	no	25 (34.7)	3 (12.00)	22 (88.00)		
RLN anastomosis (%)	yes	7 (9.7)	1 (14.29)	6 (85.71)	0.672	
	no	65 (90.3)	17 (26.15)	48 (73.85)		
Multifocality (%)	yes	25 (34.7)	6 (24.00)	19 (76.00)	1.000	
	no	47 (65.3)	12 (25.53)	35 (74.47)		
Trachea invasion (%)	yes	13 (18.1)	4 (30.77)	9 (69.23)	0.725	
	no	59 (81.9)	14 (23.73)	45 (76.27)		
Esophagus invasion (%)	yes	15 (20.8)	5 (33.33)	10 (66.67)	0.504	
	no	57 (79.2)	13 (22.81)	44 (77.19)		
Central meta (%)	yes	44 (61.1)	10 (22.73)	34 (77.27)	0.589	
	no	28 (38.9)	8 (28.57)	20 (71.43)		
N. of central meta (mean (SD))		2.10 (2.66)	3.11 (4.17)	1.76 (1.85)	0.198	
Lateral meta (%)	yes	28 (38.9)	8 (28.57)	20 (71.43)	0.589	
	no	44 (61.1)	10 (22.73)	34 (77.27)		

^†^RLN: Recurrent laryngeal nerve, IQR : Inter quartile range, SD : Standard deviation, RAI : Radioactive iodine, VCP : Vocal cord palsy.

The median follow-up time was 58 months, females were predominant, and the median patient age was 57.59 years. We also collected height, weight, and body mass index as basic information.

One patient chose to undergo hemithyroidectomy despite having T4a PTC because they wished to preserve their remaining thyroid lobes. We considered the postoperative radioactive iodine (RAI) dose as a categorical parameter, wherein 48 patients (73.8%) underwent RAI therapy with over 150 mCi and 24 patients underwent RAI therapy with less than 150 mCi.

A previous study on locally invasive thyroid cancer found that post-ablation thyroglobulin (Tg) levels that were higher than 1 contributed to a significantly increased recurrence rate^8^. In this study, the Tg levels of post-ablation varied between 1, less than 1, or more than 1. However, the correct post-ablation Tg values could not be determined in eight patients (11.1%), as RAI therapy was performed at other hospitals. Among the remaining patients, the post-ablation Tg levels were less than 1 in 34.4% of the cases and exceeded 1 in 73.8% of the cases. There was no significant association between post-ablation Tg level and PTC recurrence (*p* > 0.05).

A total of 37 patients (51.4%) had VCP before surgery and 35 patients (48.6%) did not. The RLN invasion was similar on both sides of the body, wherein the right RLN was involved in 32 patients (44.4%) and the left side was involved in 39 patients (54.1%); one patient had bilateral invasions. The median tumour size 23.28 mm, and the category value was divided into 2 cm or less and 2 cm or more. Twenty-five patients (34.7%) underwent shaving after we intraoperatively confirmed RLN invasion, and the remaining patients underwent nerve resection. Of these, we immediately performed nerve anastomoses in 7 patients. In addition to RLN involvement, 13 (18.1%) and 15 patients (20.8%) showed tracheal and esophageal invasion, respectively, while 44 (61.1%) and 28 (38.9%) patients showed central and lateral neck metastasis, respectively. The presence of central and lateral lymph node (LN) metastasis was not associated with PTC recurrence. The number of central LN metastases was not significantly different between patients with and without PTC recurrence.

After the final pathological evaluation, 39 patients (54.2%) showed clear resection margins and 33 (45.8%) showed positive/involved resection margins. Of the 39 patients with clear margins, 5 (12.82%) showed a recurrence and the remaining 34 (87.18%) did not. Of the 33 patients with involved margins, 13 (39.39%) relapsed and 20 (60.61%) showed no recurrence––this indicated a relatively high recurrence rate in the involved-margin group (*p* = 0.014). In a simple analysis of these parameters, which did not account for survival time, only the positive resection margins were statistically significant.

In the Table 2, clinico-pathological data of the resection margin positive group and the negative group were compared. As mentioned above, the recurrence rate was significantly higher in the resection margin involved group (*p* = 0.02). In addition, in the positive resection margin group, RLN resection was more frequent during surgery and the mean number of lateral LN metastases increased, which was statistically significant (*p* = 0.014 and 0.009 respectively). This is because the more aggressive the tumor is, the more lateral LN metastasis and the more RLN resection is required. As a result, the possibility of resection margin involved increase. Other variables had no statistically significant correlation with resection margin status.

**Table 2:** Comparison of the resection margin clear group with involved group.

Variable		Overall	Resection margin clear	Resection margin involved	*p* value	* *p* < 0.05
n		72	39	33		
Operative method (%)	HT	1 (1.4)	0 (0.0)	1 (3.0)	0.113	
	TT /c CND	43 (59.7)	27 (69.2)	16 (48.5)		
	TT /c CND /c LND	28 (38.9)	12 (30.8)	16 (48.5)		
Pre op. VCP (%)	yes	37 (51.4)	17 (43.6)	20 (60.6)	0.229	
	no	35 (48.6)	22 (56.4)	13 (39.4)		
Tumor size (%)	≤ 20 mm	39 (54.2)	25 (64.1)	14 (42.4)	0.109	
	> 20 mm	33 (45.8)	14 (35.9)	19 (57.6)		
RAI dose (%)	< 150	17 (26.2)	10 (28.6)	7 (23.3)	0.845	
	≥ 150	48 (73.8)	25 (71.4)	23 (76.7)		
Tg level post ablation (%)	≤ 1	22 (34.4)	15 (42.9)	7 (24.1)	0.192	
	> 1	42 (65.6)	20 (57.1)	22 (75.9)		
Intra op. RLN resection (%)	yes	47 (65.3)	20 (51.3)	27 (81.8)	0.014	*
	no	25 (34.7)	19 (48.7)	6 (18.2)		
RLN anastomosis (%)	yes	7 (9.7)	5 (12.8)	2 (6.1)	0.442	
	no	65 (90.3)	34 (87.2)	31 (93.9)		
Multifocality (%)	yes	25 (34.7)	14 (35.9)	11 (33.3)	1.000	
	no	47 (65.3)	25 (64.1)	22 (66.7)		
Trachea invasion (%)	yes	13 (18.1)	4 (10.3)	9 (27.3)	0.118	
	no	59 (81.9)	35 (89.7)	24 (72.7)		
Esophagus invasion (%)	yes	15 (20.8)	8 (20.5)	7 (21.2)	1.000	
	no	57 (79.2)	31 (79.5)	26 (78.8)		
Central meta (%)	yes	44 (61.1)	24 (61.5)	20 (60.6)	1.000	
	no	28 (38.9)	15 (38.5)	13 (39.4)		
N. of central meta (mean (SD))		2.10 (2.66)	1.79 (2.17)	2.45 (3.14)	0.313	
Lateral meta (%)	yes	28 (38.9)	11 (28.2)	17 (51.5)	0.075	
	no	44 (61.1)	28 (71.8)	16 (48.5)		
N. of lateral meta (mean (SD))		2.03 (4.04)	0.79 (1.64)	3.48 (5.39)	0.009	*
Recur (%)	yes	18 (25.0)	5 (12.8)	13 (39.4)	0.020	*
	no	54 (75.0)	34 (87.2)	20 (60.6)		

^†^VCP : Vocal cord palsy, RLN : Recurrent laryngeal nerve, ETE : Extrathyroidal extension, SD : Standard deviation.

Table 3 compares the preoperative VCP group and the intraoperative RLN-invasion group. There was no significant difference in the recurrence rates between the two groups (*p* > 0.05). There was also no difference between the two groups in terms of tumour size, resection margin, RLN anastomosis, ETE, multifocality, and tracheal or esophageal invasion (*p* > 0.05). Likewise, there was no difference between the two groups regarding the presence of central and lateral LN metastasis, and the number of lateral LN metastases. However, the mean number of central LN metastases was significantly higher in the preoperative VCP group (2.76) compared to the no-VCP group (1.40; *p* = 0.028).

**Table 3:** Comparison of the pre-operative VCP group with non pre-operative VCP group.

Variable		Overall	Pre-operative VCP	Non pre-operative VCP	*p* value	* * p* < 0.05
n		72	37	35		
Recur (%)	yes	18 (25.0)	10 (27.0)	8 (22.9)	0.788	
	no	54 (75.0)	27 (73.0)	27 (77.1)		
Tumor size (%)	≤ 20 mm	39 (54.2)	19 (51.4)	20 (57.1)	0.644	
	> 20 mm	33 (45.8)	18 (48.6)	15 (42.9)		
Resection margin (%)	clear	39 (54.2)	17 (45.9)	22 (62.9)	0.165	
	involved	33 (45.8)	20 (54.1)	13 (37.1)		
Intra op. RLN resection (%)	yes	47 (65.3)	31 (83.8)	16 (45.7)	0.001	*
	no	25 (34.7)	6 (16.2)	19 (54.3)		
RLN anastomosis (%)	yes	7 (9.7)	2 (5.4)	5 (14.3)	0.254	
	no	65 (90.3)	35 (94.6)	30 (85.7)		
ETE (%)	yes	66 (91.7)	35 (94.6)	31 (88.6)	0.423	
	no	6 (8.3)	2 (5.4)	4 (11.4)		
Multifocality (%)	yes	25 (34.7)	15 (40.5)	10 (28.6)	0.329	
	no	47 (65.3)	22 (59.5)	25 (71.4)		
Trachea invasion (%)	yes	13 (18.1)	9 (24.3)	4 (11.4)	0.222	
	no	59 (81.9)	28 (75.7)	31 (88.6)		
Esophagus invasion (%)	yes	15 (20.8)	9 (24.3)	6 (17.1)	0.566	
	no	57 (79.2)	28 (75.7)	29 (82.9)		
Central meta (%)	yes	44 (61.1)	26 (70.3)	18 (51.4)	0.147	
	no	28 (38.9)	11 (29.7)	17 (48.6)		
N. of central meta (mean (SD))		2.10 (2.66)	2.76 (3.04)	1.40 (2.00)	0.028	*
Lateral meta (%)	yes	28 (38.9)	18 (48.6)	10 (28.6)	0.095	
	no	44 (61.1)	19 (51.4)	25 (71.4)		
N. of lateral meta (mean (SD))		2.03 (4.04)	2.59 (4.21)	1.43 (3.82)	0.222	

^†^VCP : Vocal cord palsy, RLN : Recurrent laryngeal nerve, ETE : Extrathyroidal extension, SD : Standard deviation.

In the Table 3, a total of 47 patients (65.3%) underwent RLN resection and 25 did not (34.7%). In the preoperative VCP group, a majority of patients (31; 83.8%) underwent RLN resection. However, in the intraoperatively detected RLN-invasion group, the RLN was sacrificed in 16 patients (45.7%) to preserve it as far as possible. The rate of resection without preserving the RLN was relatively high in the preoperative VCP group; this result was statistically significant (*p* = 0.001).

In the univariate analysis, we found two parameters––resection margin and the number of central LN metastases––to be significant risk factors for recurrence-free survival (RFS) (*p* < 0.05; Table 4). Sex, age, height, body weight, body mass index, RAI dose, post-ablation Tg level, preoperative VCP, RLN direction, tumour size, RLN resection, RLN anastomosis, ETE, multifocality, tracheal and esophageal invasion, and lateral LN metastasis were not significantly associated with recurrence as per our statistical analysis (*p* > 0.05). In the multivariate analysis that included five parameters with *p* values of less than 0.2, we only found one independent variable––resection margin involvement––as a risk factor for RFS (hazard ratio = 3.331 [confidence interval 1.017–10.915]; *p* = 0.047).

**Table 4:** Factors predictive of recurrence by univariate and multivariate analysis.

Variable	Univariate analysis			Multivariate analysis		
	HR [95% CI]	*p* value	* * p* < 0.05	HR [95% CI]	*p* value	* * p* < 0.05
Sex = M	1.784 [0.683, 4.661]	0.237				
Age	0.999 [0.965, 1.035]	0.957				
Height	1.022 [0.965, 1.082]	0.465				
Body weight	1.024 [0.986, 1.064]	0.222				
BMI	1.070 [0.922, 1.241]	0.372				
RAI dose ≥ 150	1.084 [0.353, 3.329]	0.888				
Tg level post ablation > 1	2.759 [0.791, 9.627]	0.112		1.838 [0.494, 6.839]	0.364	
Pre op. VCP = no	0.888 [0.350, 2.253]	0.803				
RLN direction = Lt	1.221 [0.481, 3.098]	0.674				
Tumor size > 20 mm	1.626 [0.626, 4.225]	0.318				
Resection margin positive	2.982 [1.056, 8.418]	0.039	*	3.331 [1.017, 10.915]	0.047	*
Intra op. RLN resection = yes	2.530 [0.731, 8.753]	0.143		0.428 [0.091, 2.001]	0.281	
RLN anastomosis = yes	0.462 [0.061, 3.476]	0.453				
ETE = yes	1.082 [0.143, 8.168]	0.939				
Multifocality = yes	0.932 [0.349, 2.485]	0.888				
Trachea invasion = yes	1.273 [0.418, 3.876]	0.671				
Esophagus invasion = yes	1.677 [0.597, 4.715]	0.327				
Central node meta = yes	0.953 [0.373, 2.431]	0.919				
N. of central meta	1.196 [1.049, 1.364]	0.007	*	1.120 [0.992, 1.264]	0.068	
Lateral node meta = yes	1.421 [0.559, 3.611]	0.460				
N. of lateral meta	0.859 [0.701, 1.052]	0.142		0.801 [0.636, 1.008]	0.059	

^†^HR : Hazard ratio, SD : Standard deviation, RAI : Radioactive iodine, Tg : Thyroglobulin, VCP : Vocal cord palsy, RLN : Recurrent laryngeal nerve, ETE : Extrathyroidal extension.

**Figure 1:**
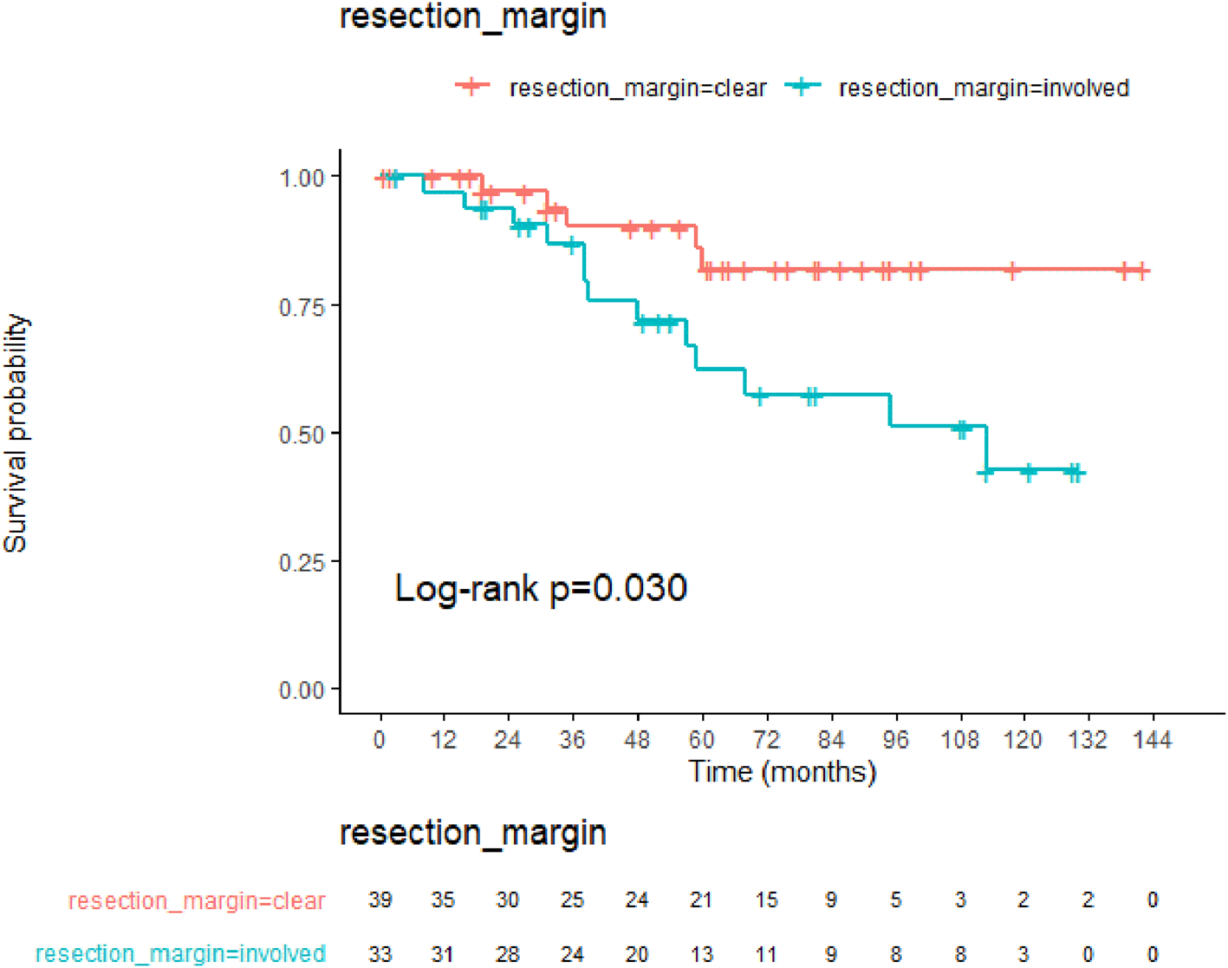
A Kaplan–Meier survival curve showing the overall recurrence-free survival of patients with a resection-margin status.

Figure 1 shows a Kaplan–Meier survival curve depicting the RFS of patients with involved resection margins. Of the 18 patients who showed recurrence during follow-up, 5 and 13 belonged to the clear- and involved-margin groups, respectively. As compared with the clear-margin group, the involved-margin group showed a significantly lower rate of RFS (82% vs. 43%; *p* = 0.03; Fig. 1).

In this study, 2 patients died because of recurrence accompanied by anaplastic change during follow-up. Since the mortality rate was so low, it was difficult to evaluate the factors associated with survival.

## Discussion

Although most patients with well-differentiated thyroid carcinomas have excellent prognoses with very low mortality rates, several staging systems for thyroid cancer have acknowledged the negative prognostic impact of local invasion^1,9^. In such cases, the death rate significantly increases to 5 ~ 35%^10^. In this regard, RLN involvement is a poor prognostic factor for T4a PTC patients. In this study, 18 patients (25%) relapsed and 2 (3%) died of anaplastic cancer after relapse. This recurrence rate was comparable to that of previous results. Chen et al.^5^ showed that recurrence occurred in 31.4% of patients in the RLN invasion group; this value was 3 times higher than that of the control group (10.3%).

RLN invasion due to thyroid cancer can be predicted by the presence of VCP before surgery^11^. However, RLN involvement is frequently identified intraoperatively. In this study, RLN invasion was confirmed in 48.6% of patients during surgery. Table 3 shows that there was no significant difference in the rate of recurrence between the preoperative VCP group and the intraoperative RLN-invasion group (27.0% and 22.9%, respectively).

The RLN may be sacrificed in cases of invasion or it can be preserved by shaving. In this study, 31 (83.8%) patients with preoperative VCP and 16 (45.7%) without preoperative VCP underwent nerve resection. Among the patients in whom the RLN was sacrificed, 14.7% underwent nerve anastomosis during surgery and 27.7% received phono-surgery (e.g., injection laryngoplasty) at follow-up. On the other hand, among 25 patients who underwent RLN shaving, 12 (48%) recovered normal vocal cord movement at the last follow-up. To date, it is controversial whether the nerve should be preserved or sacrificed in PTC patients with RLN involvement. Nishida et al.^12^ suggested that it is worthwhile to preserve the RLN, even if it is infiltrated by differentiated thyroid cancer because postoperative vocal cord function can be maintained without affecting the incidence of local recurrence or overall prognosis. Similarly, in a univariate analysis by Lang et al.^13^, RLN resection did not emerge as a significant predictor for cancer-specific survival. In our study, RLN was sacrificed intraoperatively in 47 patients (65.3%) and shaved in 25 (34.7%). This was consistent with the results of previous studies because there was no significant difference in the RFS with or without nerve resection (*p* = 0.143). Therefore, these results suggest that in cases of RLN invasion, the PTC recurrence rate is not affected by whether the nerve is resected or preserved by shaving.

In patients with of RLN invasion, the trachea and esophagus were involved in 18.1% and 20.8% of patients, respectively. These invasions were higher in the preoperative VCP group as compared to the intraoperative RLN-invasion group (24.3% vs 11.4%; 24.3% vs 17.1%), but this difference was not statistically significant. The recurrence rates of tracheal and esophageal involvement were 30.77% and 33.33%, respectively (Table 1). There was a higher recurrence rate in these patients than that of patients whose organs were not affected, but this was not a statistical difference (*p* > 0.05). These results suggest that tracheal and esophageal invasion may lead to a poor prognosis, but do not affect recurrence in patients with RLN invasion. A report by Kim et al.^8^ that included 96 locally invasive PTC patients showed results that were consistent with our study, in that they found no relationship between the recurrence rates and tracheal, laryngeal, and esophageal involvement.

The Kaplan–Meier survival curve indicated that the risk of recurrence was significantly higher in the group with positive resection margins compared to the group without positive resection margins (as per both univariate and multivariate analyses) (Table 4). Complete tumour resection is important for the prognosis of PTC patients. According to the most recent American Thyroid Association guideline, incomplete resection is a high-risk factor for tumour recurrence. Several studies on microscopic positive tumour margins have reported that there is no association with recurrence^14–16^. Recently, Abraham et al.^17,18^ reported that microscopic positive tumour margins did not affect disease-free survival in the majority of patients with PTC, but was associated with a four-fold increased risk of recurrence in T4a patients. This supports the results of our study, which was conducted among T4a patients with RLN involvement.

Several studies have investigated the association between clinical factors and the prognosis of PTC with RLN invasion. A multivariate analysis conducted by Ito et al. ^19^, found that the significant extension of PTC to other organs was strongly predictive of distant recurrence and mortality. Kim et al.^8^ reported that the post-ablation-stimulated Tg level was an independent predictor of recurrence in multivariate analysis. On the other hand, Lee et al.^20^ reported that none of the investigated factors had a significant correlation with recurrence in patients with locally invasive PTC and the exclusive involvement of a functioning RLN. In this study, the major factors affecting the prognosis of PTC patients with RLN invasion were the status of the resection margin and the number of central neck LN metastases (according to univariate analysis). In the multivariate analysis, resection margin involvement was the only factor that predisposed to a high recurrence rate. Invasion to other organs and post-ablation-stimulated Tg levels were not significant predictors of PTC recurrence. Similar studies were conducted in the past, but this is a new finding n this study that the resection margin status is a major prognostic factor in PTC patients with RLN invasion.

There are some limitations to this study. First, we did not assess the correlation between the site of diagnosis, presence of a positive margin, and the site of recurrence. Second, the status of the RLN was based on surgical findings and the decision of the surgeon, and not intraoperative neuromonitoring (IONM). A recently published guideline has promoted the use of IONM to determine whether the RLN should be preserved or sacrificed when invaded by PTC^21^. As the use of IONM in thyroid surgery has been recently limited to select cases by the Korean medical insurance system, this technique was not applied in the majority of patients. Third, the study population was relatively small. During the 14 years of this study, there were more than 3000 cases of thyroidectomy performed by this center, but only 72 patients were eligible for this study. We recommend that large-scale, multi-centre, prospective studies with a careful follow-up schedule be designed in the future to address all these limitations and further validate our results.

The prognosis of T4a PTC patients with RLN involvement was relatively poor, and the recurrence rate was not affected by preoperative VCP, intraoperative detection of RLN invasion, nerve resection, nerve preservation by shaving, LN metastasis, or tracheal or esophageal invasion. The most important prognostic factor for recurrence was a positive resection margin.

## Materials and methods

From January 2004 to December 2017, we retrospectively reviewed all patients who underwent thyroidectomy at the Department of Otorhinolaryngology, Head and Neck Surgery at the Pusan National University Hospital in Busan, Republic of Korea. During this period, 3345 patients underwent thyroid surgery at our institution. We included PTC patients with preoperative VCP or those showing post-operative VCP with intraoperatively confirmed RLN invasion. We excluded patients who presented with other types of thyroid cancer, i.e., follicular, medullary, anaplastic carcinoma, and those who had VCP due to a traction injury. Using these criteria, we considered 72 patients eligible for our study.

The patients underwent three types of surgery: hemithyroidectomy, total thyroidectomy with central neck dissection, and total thyroidectomy with central neck dissection. A majority of patients underwent a preoperative ultrasound of the thyroid, voice laboratory tests, and a thyroidectomy-related voice questionnaire. We checked the vocal movements of all patients before surgery using a laryngoscope.

The criteria for determining RLN invasion during surgery were: (1) a patient showing nerve involvement as a postoperative pathology, (2) an RLN surrounded by tumours, (3) nerves being non-identifiable due to gross extrathyroidal extension (ETE), and (4) the tumour being directly adherent to the nerve. The criteria for determining tumour recurrence were: (1) if PTC recurrence was confirmed by a postsurgical pathology following re-surgery, (2) the patient was not operated upon but showed recurrence on an ultrasound-guided biopsy performed to check suspected recurrence during the follow-up, (3) if metastatic cancer recurrence was suspected on positron emission tomography-computed tomography (CT) and chest CT.

Statistical analysis was performed using the R 3.6.2 software for Windows. Depending on whether or not normality was satisfied, we subjected the continuous variables to an independent *t*-test or Wilcoxon rank-sum test, and the categorical variables to Chi-square test or Fisher’s exact test. In the survival analysis, we used the Kaplan–Meier method to estimate the recurrence rate and the Cox proportional hazards model to test the significance.

This study was approved by the Pusan National University Hospital Institutional Review Board (IRB number: H-2004–003-089), which is organized and operates according to ICH-GCP and the applicable laws and regulations. By the Pusan National University Hospital IRB, the need for informed consent was waived for this study because the data has been provided from which personal identification information has been deleted and analysed retrospectively, such as simple charts and image reviews.

## Data Availability

The datasets are not publicly available due to protection of patient information but are available from the corresponding author on reasonable request.
